# Sensory Capacities and Eating Behavior: Intriguing Results from a Large Cohort of Italian Individuals

**DOI:** 10.3390/foods11050735

**Published:** 2022-03-02

**Authors:** Maria Pina Concas, Anna Morgan, Paola Tesolin, Aurora Santin, Giorgia Girotto, Paolo Gasparini

**Affiliations:** 1 Institute for Maternal and Child Health—IRCCS, Burlo Garofolo, 34127 Trieste, Italy; anna.morgan@burlo.trieste.it (A.M.); giorgia.girotto@burlo.trieste.it (G.G.); paolo.gasparini@burlo.trieste.it (P.G.); 2 Department of Medicine, Surgery and Health Sciences, University of Trieste, 34149 Trieste, Italy; paola.tesolin@burlo.trieste.it (P.T.); aurora.santin@burlo.trieste.it (A.S.)

**Keywords:** food adventurousness, food liking, sensory capacities, taste, smell, hearing, structural equation modelling, conditional recursive partitioning analysis

## Abstract

Eating behavior (EB) is a complex system influenced by many factors, but an undisputed role is played by the senses. In this work, we examined the effect of the sensory capacities on EB in 1152 Italian adult individuals. After administering a questionnaire on EB and assessing sensory performance through standard audiometric, olfactory, and taste tests, the prevalence of reduced sensory capacities (RSCs) and the correlation with selected risk factors were calculated. Regression models, structural equation modelling, and conditional recursive partitioning were used to investigate the relationship between variables. Around 70% of the subjects show reduced capacities in at least one sense, with taste being the most prevalent (55.21%). Male sex, aging, and low educational level are risk factors for RSCs. The increased number of senses with reduced capacities is a predictor of diminished food adventurousness and lower liking for vegetables, fish, and alcoholic beverages, while reduced capacities (RCs) in taste is a predictor of lower liking for alcoholic beverages and sweets. Overall, in addition to providing an overall picture of RSCs in Italian samples, our study reveals the association of RSCs with EB variables. This finding could have a relevant role in influencing individuals’ dietary habits and, therefore, health status.

## 1. Introduction

Eating behavior (EB) is a complex system encompassing food preferences, feeding practices, dieting, and eating-related problems including obesity, eating disorders, and feeding disorders. Food liking and food adventurousness have been shown to play a crucial role in EB, affecting food choices and intake and impacting individuals’ well-being [1]. Food liking have been described as an individual’s reported degree of liking for specific foods and beverages without regard to food intake per se [2]. It has been shown that food liking might be linked to food choices and are a proxy for reported intakes [3,4,5]. Additionally, studies have reported that food liking assessment may be a valid and feasible measure for studying the relationship between dietary behavior and health outcomes [6]. As an example, the liking for vegetables is correlated with the intake of vegetables, and it is well known that regular consumption of vegetables is linked with health benefits [7]. As regards food adventurousness (i.e., the willingness to try novel and unfamiliar foods), studies found out that the most food adventurous subjects tend to prefer a wide variety of foods, while individuals avoiding new food experiences (for example, neophobic children and adults) showed a lack of dietary variety [7,8,9,10].

Reduced taste, hearing, and smell capacities play a crucial role in EB, affecting nutrition and the perceiving of some foods [11]. For example, olfactory dysfunction affects both general perception and individual preferences of odors [12], with an impact on food selection, nutrition, and therefore health [13,14]. As a consequence, it might lead to weight loss and malnutrition, especially in the elderly [15,16]. As regards hearing loss (HL), it has been associated with low intakes of fat, protein [17], and reduced sensory input from textural cues, such as crispiness and crunchiness, leading to an increased risk of malnutrition in the elderly [18]. A lowered acuity or presence of taste loss can lead to poor appetite, inappropriate food choices, and decreased energy consumption, and it predisposes to a higher risk of developing cardiovascular diseases, overweight/obesity, and other diseases [19,20].

Based on these pieces of evidence, it is clear that reduced sensory capacities (RSCs) pose a significant threat to public health, seriously compromising the quality of life with direct consequences for health and safety and making their prevention of public interest [21,22,23,24]. Furthermore, recent findings showed that multiple RSCs exacerbate the effect of a single one [25,26], highlighting the importance of investigating reduced multisensory capacity. Several studies focused on defining the prevalence, the risk factors (e.g., age, sex, smoking habit, alcohol consumption, social status, education level, etc.), and the impact on health of single and multiple RSCs in individuals aged 40 years or more [22,27,28,29]. However, little is known about these variables and the effect of single or multiple RSCs in an entire adult population [30]. Although many studies in the literature suggest that RSCs are relatively common, there is still a lack of consensus on the prevalence of such disorders in population-based studies. In this light, the availability of phenotypic data from large cohorts of samples may offer exceptional opportunities to increase our knowledge of RSC’s prevalence and etiopathogenesis.

In this work, we analyzed a cohort of 1152 Italian adult individuals from isolated communities for which EB (food liking and food adventurousness) is available together with sensory data for hearing, smell, and taste, and personal information. The aims were to: (1) define the prevalence of single and multiple RSCs, (2) investigate the role of possible risk factors, and (3) evaluate the impact of RSCs on food liking and adventurousness.

## 2. Materials and Methods

### 2.1. Participants

Here, we analyzed part of the data collected between 2014 and 2015 from two Italian populations—Friuli Venezia Giulia (FVG), located in North-Eastern Italy, and Val Borbera (VBI), located in North-Western Italy—as a part of a research program aimed at the identification of genes and variants associated with common disorders. Inhabitants of these regions were invited to participate by public advertisements through local authorities, televisions and newspapers, and local physicians and mailings. Meetings were organized to present the project and its aims. No inclusion/exclusion criteria were applied during the recruitment phase. All the individuals, recruited as volunteers, were investigated for a large number of pathologies and phenotypes, including food habits [31] and sensory abilities on hearing, smell, and taste [32,33,34]. All parameters were systematically collected by professional and trained staff according to standardized protocols; participants were also required to fill in a questionnaire on health-related topics, including diet, lifestyle, and physical activity. All participants gave written informed consent, and the ethical committees of IRCCS-Burlo Garofolo and IRCCS-San Raffaele approved the study.

Information for a total of ~3000 individuals was collected. In this study, we applied the following inclusion criteria: (1) participation in the questionnaire about food liking and EB, (2) availability of complete information about all the three sensory phenotypes (hearing, smell, and taste), (3) availability of complete personal information (sex, age, educational attainment, smoking habits, and alcohol consumption), (4) absence of pathologies or conditions that could cause damage to senses (cancers, neurodegenerative diseases, ictus, etc.), (5) age ≥ 18 years.

### 2.2. Data Collection

For each participant, all tests and questionnaires were carried out on the same day.

#### 2.2.1. Personal and Lifestyle Characteristics

Demographic, lifestyle information, and living habits, such as cigarette smoking (current smoker/no smoker), educational attainment, and alcohol consumption (in grams/day), were collected for each participant using a standard questionnaire.

#### 2.2.2. Sensory Measurements and Reduced Sensory Capacities Definition

As regards hearing ability, all tests were performed using standard audiometers. Participants underwent pure-tone audiometry, tympanogram, and acoustic reflex testing in both ears. Measurements were all obtained after any acoustically obstructing wax had been removed. Different frequencies were measured: 0.25, 0.5, 1, 2, 4, and 8 kHz. For the statistical analysis, a new trait was created based on the mean frequency thresholds of 0.5, 1, 2, and 4 kHz, following the Bureau International d’Audiophonologie (BIAP) recommendation (http://www.biap.org/en/recommendations/65-ct-2-classification-des-surdites, accessed on: 14 December 2020). An individual was considered to have a reduced hearing capacity if the average threshold was >20.

Regarding smell, the participants were instructed about the smell testing, and they carried out the test in a dedicated room. Twelve odorants in 12 commercially available felt-tip pens (“Sniffin’ Sticks” Burghart GmbH, Wedel, Germany) [35] were presented to each participant. The olfactory test consisted of odor identification with 4-alternative forced choices. The odorants were orange, leather, cinnamon, mint, banana, lemon, licorice, coffee, clove, pineapple, rose, and fish. The number of wrong answers was counted, and reduced smell capacity was defined as the number of errors ≥ 3, similar to other studies [30,36].

Taste responsiveness was determined using the Burghart filter paper method [37]. The tastes were sweet (sucrose, 0.2 g/mL), sour (citric acid, 0.165 g/mL), salty (NaCl, 1.0 mol/L), and bitter (quinine hydrochloride, 0.0024 g/mL). Participants were tested in a dedicated room without odors or visual or auditory distractions; they were instructed by an expert administrator and were asked not to eat or brush their teeth beforehand. Specifically, each participant placed the paper on his/her tongue and had to recognize the correct taste among a list of four descriptors: sweet, bitter, salt, and sour. A possible choice was also “I do not perceive any taste”. After tasting each paper, the participant rinsed his/her mouth with 10 mL of bottled mineral water (room temperature). The number of errors was counted, including as an error the answer “I do not perceive any taste”. Reduced taste capacity was defined as the number of errors ≥ 1, in agreement with other studies [28,30,36].

Each sensory capacity variable was coded as a binary trait considering the presence (1) and absence (0) of a reduced threshold. The number of the senses with reduced capacities was coded as an ordinal variable.

#### 2.2.3. Eating Behavior

Liking of about 100 foods and beverages was assessed through a questionnaire. For each food, the participants answered the question “How much do you like it?” and they rated the preference on a 9-point scale ranging from “dislike extremely” (score 1) to “like extremely” (score 9) [38]. The liking groups were defined as the average of likings given by each individual for specific foods, and seven groups were taken into account as already described in [31]: alcoholic beverages, cheeses, fish, fruit, meat, sweet foods, and vegetables.

Food adventurousness was assessed as explained in [31]. Briefly, each subject answered the question: “How often do you try unfamiliar foods?” The response categories were: “never”, “rarely”, “some of the time”, “often”, and “very often”, categorized for the statistical analysis as numbers from zero to four.

### 2.3. Statistical Analysis

Statistical analyses were performed in R 3.6.2. (www.r-project.org, accessed on: 14 December 2020). The significance criterion was *p* ≤ 0.05.

Preliminary analysis conducted in each population separately (data not shown) revealed a similar trend, and we decided to merge all subjects in a unique dataset.

The prevalence of RSCs, calculated as a percentage (i.e., prevalence proportion), was described as a whole and according to sex and age groups.

The relationships between independent variables as possible risk factors and RSCs were assessed through logistic regression models for each sense function and through ordinal regression model (polr function, MASS R library) for the number of RSCs, which was coded as an ordinal variable from zero to three. The explanatory variables set included sex, age, education, smoking habits, and alcohol consumption. Age was considered in 10-year groups to capture better the effect. Education was used as a categorical variable: low (elementary/lower secondary) versus high (upper secondary/university). According to previous studies, alcohol consumption was classified into two categories (i.e., low and high) [30].

Multiple regression models were conducted to evaluate the possible effect of the number of RSCs on the EB phenotypes, i.e., food liking and food adventurousness in the entire population. Moreover, to better understand the complex interplay between variables, models were developed and tested with structural equation modelling (SEM, lavaan R package [39]). SEM [40] is a statistical technique that identifies the direct and indirect influences of variables. It is used when the response variable in one regression equation becomes a predictor in another. Potential confounders (sex, age, and educational level) were included in the models. Criteria for overall fit were chosen a priori: chi-square *p*-value non-significant (χ^2^ *p* > 0.05), confirmatory fit index (CFI) ≥ 0.92, Tucker–Lewis Index (TLI) > 0.87, and root mean square error of approximation (RMSEA) < 0.05 [41]. Finally, to evaluate the significance of the mediated model, the Sobel test was used [42]. In the statistical analysis, food adventurousness was considered as a numeric score ranging from 0 (“never”) to 4 (“very often”) and studied as a quantitative trait.

Conditional inference tree analysis was used to verify the effect of the number of RSCs on EB variables, in combination with the information on impairment in taste, hearing, and smell. Indeed, this method is not affected by multicollinearity and could be used also in presence of high correlations between independent variables, such as sensory capacity variables in our sample. We used the ctree function of R’s Partykit package (https://cran.r-project.org/web/packages/partykit/vignettes/partykit.pdf, accessed on: 14 December 2020). This statistical method [43] performs recursive partitioning that allows the creation of a regression tree; the variable with the highest predictive power (the lowest *p*-value after Bonferroni correction) is represented as the first node in the decision tree, so two subgroups (I and II) are created. For subgroup I, the variable with the lowest *p*-value (if any) is taken as the second or third node. The same is done for subgroup II. The final model is based on the splitting variables in each node with the highest statistical significance [44].

## 3. Results

### 3.1. Sample Characteristics

A total of 1152 individuals (710 from Friuli Venezia Giulia (FVG) and 442 from Val Borbera (VBI)) aged 18–89 years (mean age 51.6 ± 16.6) were included in the study. Sample features, considering all the individuals and their sensory impairment status, are summarized in Table 1.

Table S1 shows, for the whole population and according to age groups, the mean and standard deviation of each sensory variable (mean frequency thresholds of 0.5, 1, 2, and 4 kHz for hearing and the number of errors for both smell and taste) and for each EB variable (food adventurousness and food liking).

As regards smell, based on the number of correct answers in the Sniffin’s stick test [35], in our population, the number of normosmic individuals (≥11 correct answers) was 691 (60.0%), the number of hyposmics (between 8 and 10 correct answers) was 409 (35.5%), and the number of anosmics (≤7 correct answers) was 52 (4.5%).

### 3.2. Prevalence of Reduced Sensory Capacities and Factors Influencing Sensory Ability

**Figure 1:**
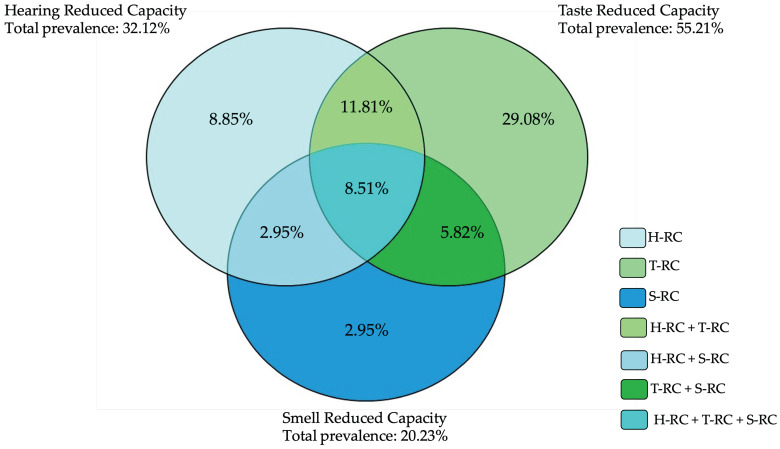
Venn diagram of the prevalence of reduced sensory capacities (RSCs). The graph shows the distribution of RSCs in our populations, highlighting the prevalence of single and multiple RSCs. H-RC, T-RC, S-RC = reduced capacity in hearing, taste, and smell, respectively.

Figure 1 shows a Venn diagram of the prevalence of RSCs in our populations.

Considering the whole population, 346 out of 1152 (30.03%) subjects did not show any sense with RC. One sense with RC was present in 40.88% of the sample (8.85% hearing, 29.08% taste, and 2.95% smell), two in 20.58% (11.81% hearing and taste, 5.82% taste and smell, 2.95% hearing and smell), and three in 8.51%. Taste RC was the most common (overall 55.21% and 29.08% as single sense), followed by hearing (overall 32.12% and 8.85% as single) and smell (overall 20.23% and 2.95% as single one). Taste plus hearing was the most frequent combination of two senses with RCs (11.81%), while the less frequent was hearing plus smell (2.95%). Table S2 shows the number and percentage of individuals presenting one sense with RC (i.e., in hearing, smell, or taste), two and three RSCs, in all samples and according to age groups. In all the age groups, the single RC in taste was the most frequent, except in 70+ people, in which the concurrent RCs in the three senses was the most frequent (35%).

**Figure 2:**
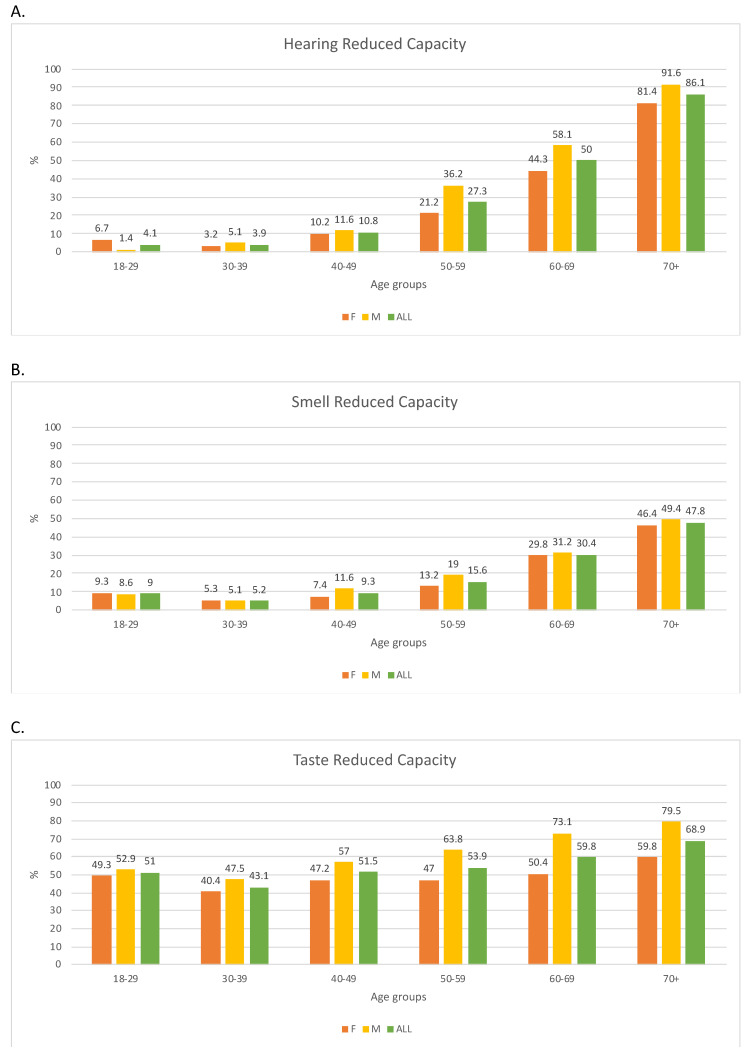
Prevalence of reduced capacity for hearing (**A**), smell (**B**), and taste (**C**) in the 10-year age groups for females (orange bars), males (yellow bars), and overall (green bars). The *y*-axis indicates the age groups, and the *x*-axis presents the prevalence as a percentage. The numbers above the bars are the prevalence.

The prevalence of hearing, smell, and taste RC in females, males, and overall samples by 10-year age groups is shown in Figure 2.

In both sexes, starting from the age of 30, the prevalence of hearing RC almost doubled every decade. In the vast majority (>80%), hearing RC was very common in the elderly (age 70+). From the age of 30–39, its prevalence in males was higher than in females, although this difference was statistically significant only in the age group 50–59 (χ^2^ test *p*-value 0.01). The prevalence of smell RC reached 20% in the decade 60–69 and almost 50% in the 70+ group. No significant differences were found between the sexes (χ^2^ test *p*-value > 0.05). Regarding taste, the prevalence ranged from 40.4% (women 30–39) to 79.5% (men 70+). The prevalence in males was always higher than females, and it was statistically significant in age groups 50–59 and later (χ^2^ test *p*-value < 0.01).

**Figure 3:**
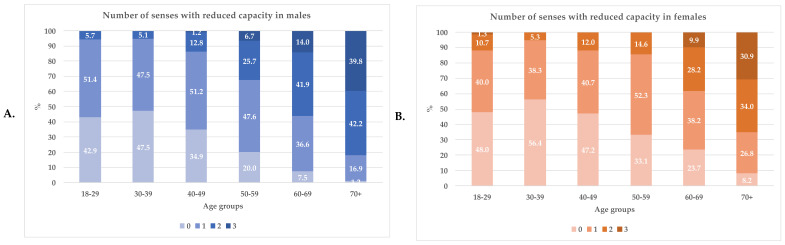
Prevalence of the number of senses with reduced capacities in males (**A**) and females (**B**) by 10-year age groups. The numbers above the bars are the prevalence. The colors indicate the number of senses with reduced capacities: the absence of sense with RC is indicated by the lighter color, the presence of three senses with RCs by the darkest.

Figure 3 shows the prevalence of single/multiple RSCs divided by age groups in females and males.

The proportion of people without RSCs added to subjects with only one sense with RC was about 90% in the younger population (<40 years) and decreased to 35% and ~18% in women and men over 70. The proportion of single RC was about 40% in women and 50% in men up to the decade 50–59, after which it further decreased, with lower values for males but with no statistical differences between the sexes. Conversely, the percentage of two and three rose between age groups to 82% in 70+ males and 64.9% in 70+ females. The prevalence of three RSCs was higher in males, and the difference between genders was statistically significant for the age group 50–59 y (*p*-value < 0.01).

Table S3 shows the association of sex, age, low education, high alcohol consumption, and smoking with the different senses with RCs and their number.

As already observed in terms of prevalence, gender (i.e., male) was a significant risk factor for hearing and taste RCs (OR 1.78 and 1.9, respectively, *p*-value < 0.001) and for the overall number of senses with RCs (OR 1.91, *p*-value < 0.0001). All sensory abilities were affected by aging: a 10-year increase in aging produced an increased risk for taste (1.13), for smell (1.67), and hearing ability (3.08, *p*-values < 0.01). In addition, a 10-year increase in aging corresponded to an increase in the risk of 1.81 in the number of senses with RCs (*p*-value < 0.0001). Low education level was a risk factor for smell, taste (OR 1.62 and 1.48, respectively, *p*-values < 0.01), and the overall number of senses with RCs, with an OR of 1.67 (*p*-value < 0.0001). We found no significant influences from smoking and high alcohol consumption.

### 3.3. Effect of Reduced Sensory Capacities on Food Liking and Food Adventurousness

All the liking groups showed good reliability (Cronbach alpha ≥ 0.73 in FVG and VBI).

**Figure 4:**
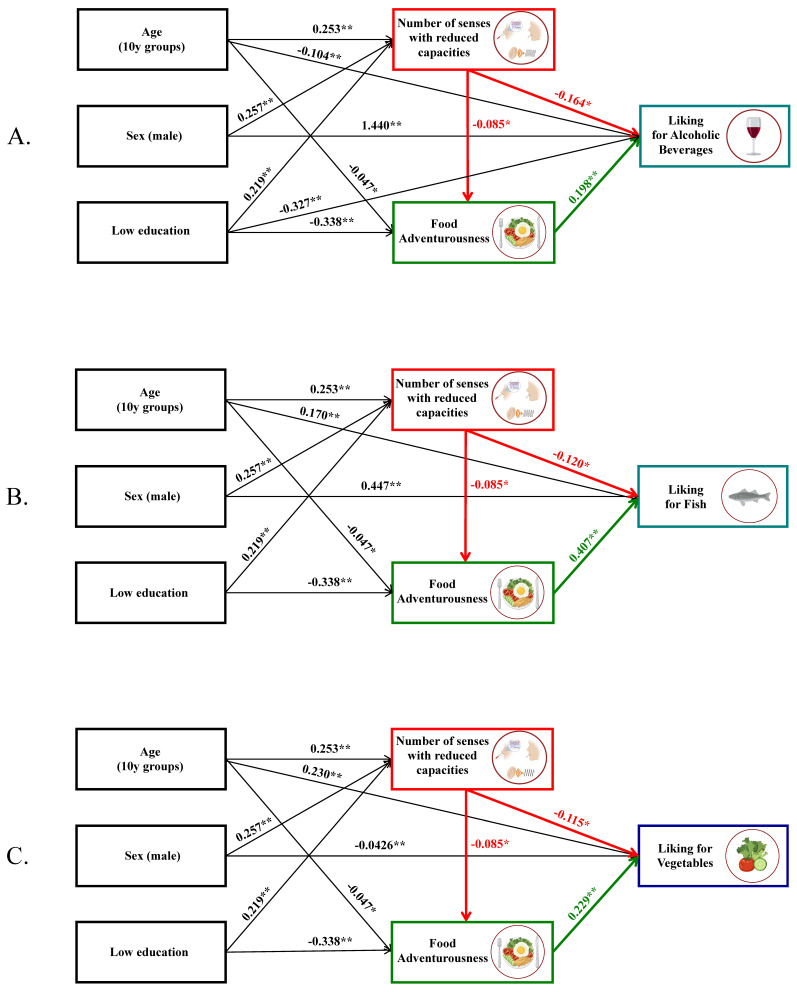
Structural equation models (Lavaan R package [39]) show the effect of the number of senses with reduced capacities on food adventurousness and liking for alcoholic beverages (**A**), fish (**B**), and vegetables (**C**). The models have a good fit (CFI = 0.998, TLI = 0.978, *p*-value chi-square = 0.134, RMSEA = 0.033 for alcoholic beverages; CFI = 0.998, TLI = 0.973, *p*-value chi-square = 0.134, RMSEA = 0.033 for fish; and CFI = 0.999, TLI = 0.995, *p*-value chi-square = 0.286, RMSEA = 0.015 for vegetables). Reported numbers are the effect of the independent variables on the dependent variables. * *p* value < 0.05, ** *p*-value < 0.001. y = years.

Table S4 shows the effect of the number of senses with RCs on liking groups and food adventurousness. A higher number of senses with RCs was a significant predictor of a lower preference for alcoholic beverages, fish, and vegetables (*p*-value 0.01, 0.04, and 0.009, respectively). Additionally, a higher number of senses with RCs corresponded to lower values of food adventurousness, but the statistical significance was at the threshold (*p*-value 0.05). Because all the liking groups were associated with food adventurousness (Table S3), the effect of the RSCs on the preference for alcoholic beverages, vegetables, and fish could be mediated by food adventurousness. Therefore, structural equation modelling (SEM) and Sobel tests were performed to examine the direct and mediated effect of variables on the liking groups. A direct effect of the number of senses with RCs on the liking groups emerged in complex models, with interaction with gender, age, and education level (Figure 4A–C). The Sobel test revealed that the effect of RSCs on food liking groups was also mediated by food adventurousness for alcoholic beverages (z = −3.38, *p*-value = 0.001), for fish (z = −4.72, *p*-value < 0.001), and for vegetables (z = −3.96, *p*-value < 0.001). The SEM models had good fit parameters (CFI = 0.998, TLI = 0.978, *p*-value chi-square = 0.134, RMSEA = 0.033 for alcoholic beverages; CFI = 0.998, TLI = 0.973, *p*-value chi-square = 0.134, RMSEA = 0.033 for fish; and CFI = 0.999, TLI = 0.995, *p*-value chi-square = 0.286, RMSEA = 0.015 for vegetables).

**Figure 5:**
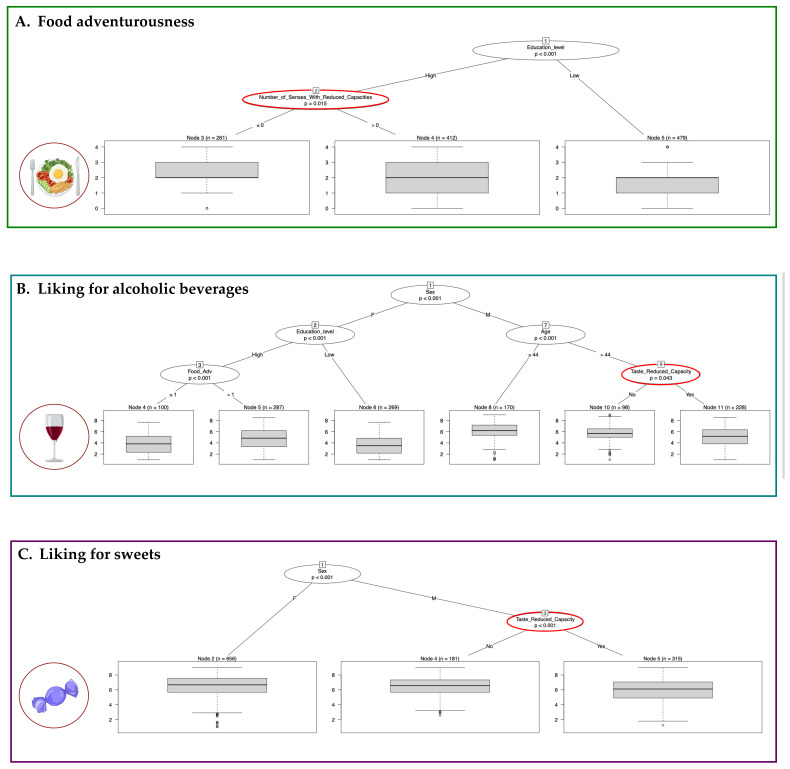
Binary trees, computed by conditional recursive partitioning, of the effect of the number of senses with reduced capacities and reduced capacity in hearing, smell, and taste, together with gender, age, food adventurousness, and education level, on food adventurousness (**A**), liking for alcoholic beverages (**B**), and liking for sweets (**C**).

To better understand the role of the RSCs on EB measures, we applied conditional recursive partitioning. In Figure 5 are shown the binary trees obtained for food adventurousness and for the liking for alcoholic beverages and sweets in which the effects of the number of senses with RCs and single sense with reduced capacity were detected, while Figure S1 shows the binary trees obtained for the other food liking in which the effect of RSCs was not found.

Considering food adventurousness as a dependent variable (Figure 5A), we found that the analysis was able to divide the whole sample into two subgroups according to high/low education level (node 1, *p*-value < 0.001). The subgroup with high education level was further split according to the number of senses with RCs (node 2, cutoff 0, *p*-value = 0.015). Considering a boxplot that indicated the distribution of food adventurousness in each subgroup, we can conclude that the individuals with low education (node 5, *n* = 479) had lower values of the trait, followed by individuals with high education but at least one sense with reduced capacity (node 4, *n* = 412) and by individuals with high education and normal sensory capacity (node 3, *n* = 261).

As regards the liking for alcoholic beverages (Figure 5B), the reduced capacity for taste divided the males aged 44 years and above into two subsamples (node 9, *p*-value 0.043). Subjects with RC (node 11, *n* = 228) showed a lower liking compared with those without RC (node 10, *n* = 98). Other influencing variables were education level and food adventurousness.

Finally, only taste RC affected the liking for sweets (Figure 5C, node 3, *p*-value < 0.001). In particular, males with RC (node 5, *n* = 315) showed lower liking compared with those without (node 4, *n* = 181).

## 4. Discussion

Single and multiple RSCs play a critical role in EB and, in general, in the quality of life, being even more relevant in the elderly. In this work, we report the data on their prevalence and association with possible risk factors in a large Italian cohort and their link with EB.

Regarding the prevalence of RSCs in our population, taste RCs are the most frequent ones, followed by hearing and smell. About 70% of individuals had at least one sense with RC, consistent with the data already reported for other countries, such as Germany (with a prevalence of one or more RSCs in 73.6% of the subjects [30]). Conversely, some differences were found with the prevalence of a single sense with RC; in the German population, the hearing RC was slightly higher (43.9%), while that of taste was lower (20.3%) than our Italian data. Analyzing a population of approximately 3500 American subjects aged 40 and over, Liu et al. (2016) [27] found a prevalence of smell RC (13.5%) comparable with ours, while the percentage of taste RC was lower (17.3%). In another study of approximately 3000 American individuals, the results were comparable with ours for taste RC but lower for the remaining two senses [28]. In principle, the difference with other studies could be due to the population’s specific features (i.e., different age range, ethnicity, etc.) or to the different methodology used to assess each sense capacity. For example, the hearing ability was assessed by subjective means, such as interviews in Correia et al. (2016) [28], but by instrumentation (i.e., objective means such as audiometric evaluation) in Khil et al. (2015) [30] and our study. Instrumental data represent a more precise way of calculating hearing thresholds, taking into consideration several audiometric frequencies. Regarding the smell ability, despite being evaluated in all studies in a more harmonized way using the Sniffin’ Sticks test, the number of odorants tested varies greatly study by study, ranging from 8 [27] to 12 in our study. Finally, to measure taste ability, Correia and colleagues (2016) [28] used the same filter papers we utilized, while Khil (2015) [30] adopted spray bottles.

Regarding possible risk factors for RSCs, we found that male gender was associated with a higher prevalence of hearing, taste, and multiple RCs, in accordance with previous studies [27,28,30]. Sex differences might be due to genetic factors, lifestyle-related diseases, and different attention to health and work exposure [45]. As expected, we observed that, with aging, all the senses worsen, and the number of senses with RCs increases. Indeed, it is already known that the aging process is associated with a decline in sense function, and possible explanations could be physiological and anatomical changes during life and accumulations of specific lifestyles or exposures over time with aging [11,23,46]. We found associations of RSCs with a low educational level, a socio-economic indicator, and then with a proxy of a healthy lifestyle and different exposure to environmental risk factors. In this light, our results are consistent with Khil et al. (2015) [30], whereas other studies reported an association of low educational level only with smell but not taste [27]. The role of smoking habits and high alcohol consumption was largely debated but controversial [27,30], and our findings exclude any possible role of these two attitudes in the populations under investigation.

As regards the effect of RSCs on EB variables, our data show that a higher number of senses with RC was associated with lower food adventurousness and liking for some foods, such as alcoholic beverages, vegetables, and fish. In addition, by means of conditional recursive partitioning, we were able to confirm the effect of the number of senses with RC on food adventurousness and to highlight the effect of taste RC on liking for alcoholic beverages and sweets in particular subsamples of individuals. To our knowledge, no other studies considered the effect of concurrent RSCs on EB. As regards RC in taste, which we found specifically associated with the liking for alcoholic beverages and sweets, many researchers investigated the role of the capacity to perceive a single taste (i.e., bitter or sweetener) on liking but controversial results are present [47,48,49,50,51]. Studies have investigated the effect of a single sense with reduced capacity in food liking. For example, Zang and colleagues found that the liking for chocolate and peanut was lower in individuals with smell dysfunctions compared with controls [52].

Since food liking could be considered a measure of food intake [31,53] and therefore correlated with diet, our data further reinforce the role of RSCs in EB. A lower food adventurousness in the elderly has already been described as a sign of low dietary variety and quality [54,55]. Other studies showed that people with RSCs have a poor diet. For example, individuals with smell dysfunctions have a reduced preference for fruits and vegetables [56] or consume a low-quality diet [57]. Other studies showed that food neophobia (i.e., the reluctance to try novel foods, almost the opposite measure of food adventurousness) is associated with chemosensory abilities such as smell and taste [58], in line with our findings.

Furthermore, our work clearly further demonstrates how complex the relationship is between sensory abilities and diet-related phenotypes. This aspect suggests that many factors must be included in this kind of study and that appropriate statistical instruments—such as, for example, structural equation modelling or conditional recursive partitioning analysis—are needed.

The present study has some limitations, here addressed. First, it did not include data about food intake, and therefore, no direct effect of RSCs on consumption could be evaluated. In addition, it the difference between our hearing and chemosensory tests deserve to be mentioned. Indeed, the test we used to assess hearing capacity was a detection test, i.e., it measured the ability of participants to detect sounds from various frequencies. On the contrary, our taste and smell tests were forced-choice identification, i.e., they assessed the ability of the participants to identify different tastes and odors. The identification process involves the ability to perceive or detect sensory stimuli and other factors, such as verbal capacity and knowledge of the specific odorants and tastes. Indeed, chemosensory identifications rely on semantic and episodic memory functions and depend on encoding, retrieval, and verbal mediation processes [59,60,61,62]. In particular, since these processes are age-sensitive, an important factor underlying age-related differences in odor/taste identification can be the efficiency with which verbal representations of odors/tastes are retrieved [61,63]. Researchers have proposed specific tests for exploring the variability in chemosensory abilities within a population of older adults, including frail and dependent individuals [63,64]. In these experiments, taste capacity was measured by a detection test in which verbal ability and memory recovery were not required. These tests are not easy to implement in general population surveys such as our work. Still, it is crucial to consider these issues in future studies in order to obtain results for chemosensory tests that more comparable with hearing tests. Considering these aspects, we did not use the term “sensory deficit” or “sensory impairment” employed in other studies. We chose “reduced sensory capacity”, which allowed us to include the two dimensions assessed in our identification tests, i.e., the ability to detect the odorous/taste compounds and the ability to recover the name of the corresponding odor/taste.

Another limitation is the choice of the cut-offs used to determine the reduced sensory capacities. Indeed, there are no specific guidelines, and we relied on similar studies [28,30,36]. In addition, we observed a high prevalence of reduced capacity for taste in our results. This is probably due to the definition of reduced capacity being whether the participant identified three tastes out of four, as already proposed in other studies [28,30]. In addition, it deserves mentioning the phenome of “taste confusion”, i.e., the fact that many people misidentify the quality of taste stimuli, particularly evident for bitter and sour taste [65]. This aspect is due to age and other factors such as smoking and head trauma [65]. All these considerations give an idea of how difficult it is to determine the “true” prevalence of reduced sensory capacities, which is an issue that must be considered in future studies.

## 5. Conclusions

Overall, the present findings provide a unique picture of the prevalence of RSCs in a Caucasian population. The frequent presence of multiple RSCs (~30%) further strengthens the hypothesis that there could be a common pathophysiology among the senses as well as shared environmental and lifestyle risk factors [25,30,46].

Furthermore, for the first time, it was demonstrated that there is a correlation between an increased number of RSCs and a reduced food adventurousness and liking for specific foods, with possible consequences on the individuals’ dietary habits and, therefore, their health status. In this light, any intervention aimed at the preservation of sensory function is essential since it could have a huge impact not only on the overall quality of life but also on food preference and intake, thus impacting individual health status.

## Figures and Tables

**Table 1:** Characteristics of study participants, considering all the individuals and according to sensory status (i.e., no sense with reduced capacity and at least one sense with reduced capacity). IQR = interquartile range; Food adv = food adventurousness.

	All	No Sense with Reduced Capacity	At Least One Sense with Reduced Capacity
N (% males)	1152 (43.1)	346 (33.8)	806 (47.0)
Age groups, N (%)			
18–29	145 (12.6)	66 (19.1)	79 (9.80)
30–39	153 (13.3)	81 (23.4)	72 (8.93)
40–49	194 (16.8)	81 (23.4)	113 (14.0)
50–59	256 (22.2)	71 (20.5)	185 (23.0)
60–69	224 (19.4)	38 (11.0)	186 (23.1)
70+	180 (15.6)	9 (2.60)	171 (21.2)
Education, N (%)			
Elementary	170 (14.8)	17 (4.91)	153 (19.0)
Lower secondary	309 (26.8)	68 (19.7)	241 (30.0)
Upper secondary	516 (44.8)	182 (52.6)	334 (41.4)
University	157 (13.6)	79 (22.8)	78 (9.68)
Current smokers, N (%)	224 (19.4)	80 (23.1)	144 (17.9)
High alcohol consumers, N (%)	298 (25.9)	79 (22.8)	219 (27.2)
Food adv, median (IQR)	2.0 (1; 3)	2.0 (1.3; 3.0)	2.0 (1.0; 3.0)
Liking groups, median (IQR)			
Alcoholic beverages	4.8 (3.3; 6.2)	5.0 (3.7; 6.5)	4.8 (3.2; 6.2)
Cheeses	6.3 (5.3; 7.0)	6.3 (5.3; 7.2)	6.2 (5.3; 7.0)
Fish	6.7 (5.3; 7.5)	6.8 (5.5; 7.5)	6.5 (5.3; 7.3)
Fruit	7.0 (6.3; 7.7)	7.0 (6.4; 7.7)	7.0 (6.3; 7.7)
Meat	6.8 (6.1; 7.5)	7.0 (6.0; 7.6)	6.8 (6.1; 7.5)
Sweets	6.4 (5.4; 7.3)	6.6 (5.6; 7.3)	6.4 (5.3; 7.3)
Vegetables	6.7 (5.9; 7.5)	6.8 (5.9; 7.6)	6.7 (5.9; 7.5)

## Data Availability

The data presented in this study are available on request from the corresponding author. The data are not publicly available due to privacy restrictions.

## Supplementary Materials

The following supporting information can be downloaded at: https://www.mdpi.com/article/10.3390/foods11050735/s1, Table S1: mean and standard deviation of each sensory variable (mean frequency thresholds of 0.5, 1, 2, and 4 kHz for hearing and the number of errors for both smell and taste) and for each EB variable (food adventurousness and food liking), Table S2: number and percentage of individuals presenting one sense with reduced capacity (RSC) (i.e., in hearing, smell, or taste), two and three RSCs, according to age groups, Table S3: Factors associated with hearing, smell, and taste abilities (tested by logistic regression analysis) and the numbers of senses with reduced capacity (tested by ordinal logistic regression; the categories are the number of the senses with reduced capacity), Table S4: Results of linear regression models for assessing the relationship between RSC and eating behavior, Figure S1: Binary trees, computed by conditional recursive partitioning, of the effect of the number of senses with reduced capacity and RC in hearing, smell, and taste, together with gender, age, food adventurousness, and education level, on liking for meat (A), vegetables (B), cheeses (C), fruit (D), and fish I. Only gender, age, and food adventurousness affected the liking for these foods.
