# Supplementary material for: Sensory Capacities and Eating Behavior: Intriguing Results from a Large Cohort of Italian Individuals
Source: Foods. 2022 Mar 2;11(5):735. doi: 10.3390/foods11050735 (PMC8909480; doi:10.3390/foods11050735)
Supplement: Supplementary file 1 [file foods-11-00735-s001.zip › FigureS1.pdf]

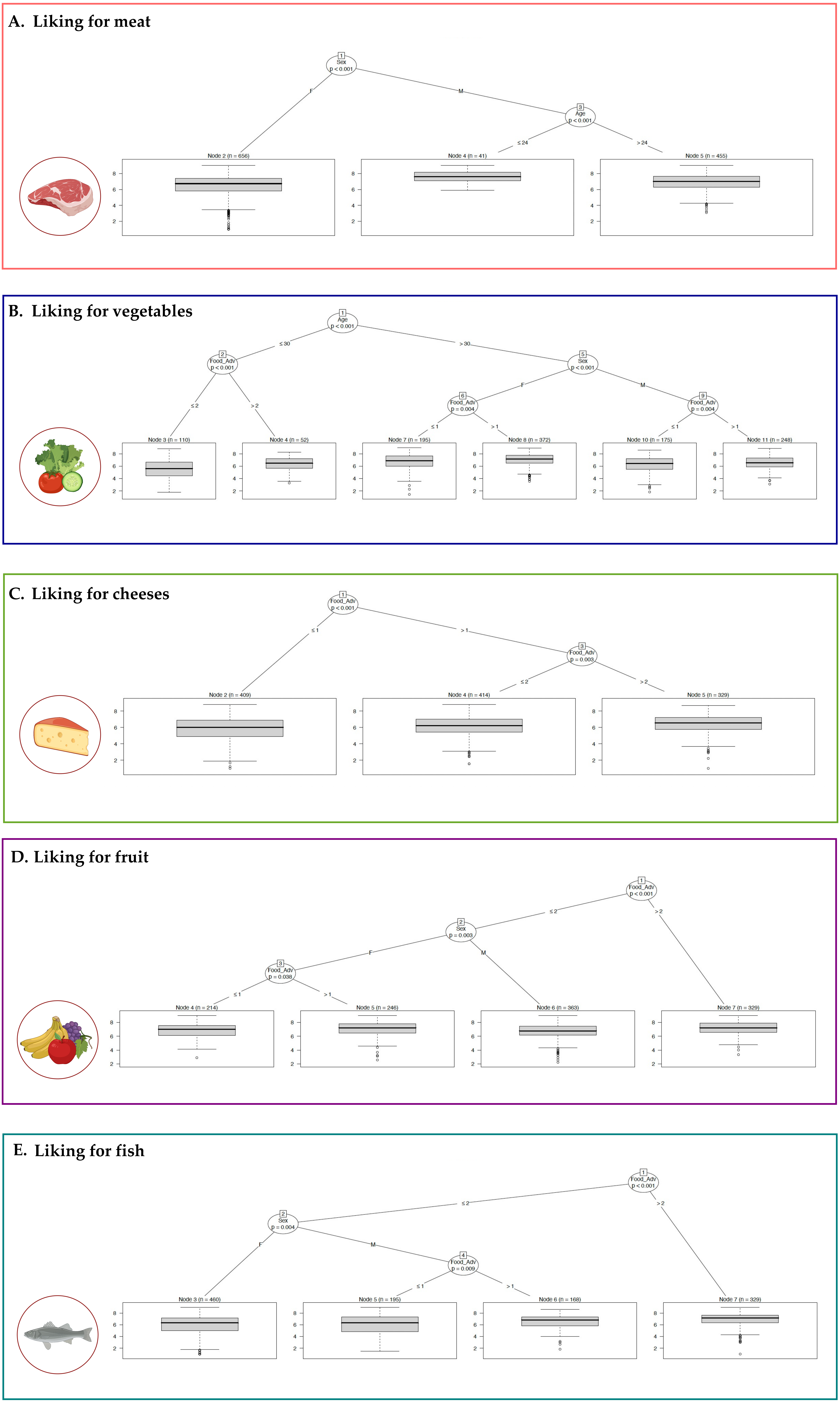
A. Liking for meat
1
Sex
p < 0.001
F
M
3
Age
p < 0.001
≤ 24
> 24
Node 2 (n = 656)
Node 4 (n = 41)
Node 5 (n = 455)
B. Liking for vegetables
1
Age
p < 0.001
≤ 30
> 30
2
Food_Adv
p < 0.001
≤ 2
> 2
5
Sex
p < 0.001
F
M
6
Food_Adv
p = 0.004
≤ 1
> 1
9
Food_Adv
p = 0.004
≤ 1
> 1
Node 3 (n = 110)
Node 4 (n = 52)
Node 7 (n = 195)
Node 8 (n = 372)
Node 10 (n = 175)
Node 11 (n = 248)
C. Liking for cheeses
1
Food_Adv
p < 0.001
≤ 1
> 1
3
Food_Adv
p = 0.003
≤ 2
> 2
Node 2 (n = 409)
Node 4 (n = 414)
Node 5 (n = 329)
D. Liking for fruit
1
Food_Adv
p < 0.001
≤ 2
> 2
2
Sex
p = 0.003
F
M
3
Food_Adv
p = 0.038
≤ 1
> 1
Node 4 (n = 214)
Node 5 (n = 246)
Node 6 (n = 363)
Node 7 (n = 329)
E. Liking for fish
1
Food_Adv
p < 0.001
≤ 2
> 2
2
Sex
p = 0.004
F
M
4
Food_Adv
p = 0.009
≤ 1
> 1
Node 3 (n = 460)
Node 5 (n = 195)
Node 6 (n = 168)
Node 7 (n = 329)
