# Supplementary material for: Sensory Capacities and Eating Behavior: Intriguing Results from a Large Cohort of Italian Individuals
Source: Foods. 2022 Mar 2;11(5):735. doi: 10.3390/foods11050735 (PMC8909480; doi:10.3390/foods11050735)
Supplement: Supplementary file 1 [file foods-11-00735-s001.zip › Supplem_Tables.pdf]

Table S1: Mean and standard deviation of each sensory variable (mean frequency thresholds of 0.5, 1, 2, and 4 kHz for hearing and the number of errors for both smell and taste) and for each EB variable (food adventurousness and food liking). Food adv: food adventurousness.

Age group	Hearing (mean frequency thresholds of 0.5, 1, 2, and 4 kHz)	Taste (number of errors)	Smell (number of errors)	Food adv	Liking for alcoholic beverages	Liking for cheeses	Liking for fish	Liking for fruit	Liking for meat	Liking for sweet	Liking for vegetables
**All**	18.95 (13.02)	0.76 (0.83)	1.48 (1.49)	1.99 (1.05)	4.67 (1.93)	6.09 (1.3)	6.29 (1.61)	6.93 (1.09)	6.67 (1.31)	6.29 (1.5)	6.6 (1.26)
**18-29**	9.08 (7.54)	0.72 (0.86)	1.08 (1.1)	2.16 (0.93)	5.3 (1.87)	6.08 (1.18)	5.98 (1.75)	6.77 (1.24)	7.03 (1.27)	6.56 (1.22)	5.79 (1.5)
**30-39**	9.91 (6.83)	0.54 (0.69)	0.83 (0.97)	2.29 (0.96)	5.07 (1.86)	5.99 (1.46)	6.21 (1.65)	7.00 (0.91)	6.67 (1.35)	6.35 (1.39)	6.5 (1.19)
**40-49**	13.45 (5.87)	0.68 (0.79)	1.05 (1.17)	2.16 (1.03)	4.81 (2.01)	6.07 (1.23)	6.25 (1.68)	7.03 (1.02)	6.52 (1.4)	6.28 (1.4)	6.58 (1.26)
**50-59**	17.42 (8.48)	0.7 (0.77)	1.3 (1.26)	1.97 (1.05)	4.59 (1.97)	6.09 (1.35)	6.48 (1.51)	7.02 (1.09)	6.54 (1.31)	6.25 (1.66)	6.79 (1.21)
**60-69**	24.2 (12.02)	0.83 (0.85)	1.8 (1.49)	1.87 (1.12)	4.31 (1.91)	6.17 (1.31)	6.44 (1.57)	6.92 (1.08)	6.73 (1.41)	6.21 (1.57)	6.83 (1.13)
**70+**	36.17 (12.94)	1.04 (0.92)	2.66 (1.92)	1.61 (1.02)	4.21 (1.72)	6.12 (1.24)	6.19 (1.53)	6.78 (1.19)	6.67 (1.04)	6.19 (1.53)	6.78 (1.09)

Table S2: number and percentage of individuals presenting one sense with reduced capacity (i.e., in hearing, smell, or taste), two and three, according to age groups.

		Reduced capacity						
**Age group, N (%)**	**No sense with reduced capacity**	**Taste**	**Smell**	**Hearing**	**Hearing+Taste**	**Hearing+Smell**	**Taste+Smell**	**Hearing+Taste+Smell**
**All**	346 (30.0)	335 (29.1)	34 (2.95)	102 (8.85)	136 (11.8)	34 (2.95)	67 (5.82)	98 (8.51)
**18-29**	66 (45.5)	61 (42.1)	4 (2.76)	1 (0.69)	4 (2.76)	0 (0.00)	8 (5.52)	1 (0.69)
**30-39**	81 (52.9)	58 (37.9)	4 (2.61)	2 (1.31)	4 (2.61)	0 (0.00)	4 (2.61)	0 (0.00)
**40-49**	81 (41.7)	76 (39.2)	4 (2.06)	8 (4.12)	11 (5.67)	1 (0.51)	12 (6.18)	1 (0.51)
**50-59**	71 (27.7)	85 (33.2)	12 (4.69)	32 (12.5)	28 (10.9)	3 (1.17)	18 (7.03)	7 (2.73)
**60-69**	38 (17.0)	47 (21.0)	8 (3.57)	29 (12.9)	42 (18.7)	15 (6.70)	19 (8.48)	26 (11.6)
**70+**	9 (5.00)	8 (4.44)	2 (1.11)	30 (16.7)	47 (26.1)	15 (8.33)	6 (3.33)	63 (35.0)

Table S3. Factors associated with hearing, smell, and taste abilities (coded as 0 = normal capacity and 1 = reduced capacity, and tested by logistic regression analysis) and the numbers of senses with reduced capacity (tested by ordinal logistic regression; the categories are the number of senses with RC). The values are Odds Ratio (OR) and 95% Confidence Interval (CI). In brackets is reported the p-value of the association. In bold are reported significant associations (p-value<0.05).

**OR 95%CI (p-value)**	**Hearing**	**Smell**	**Taste**	**Number of senses with reduced capacity**
Sex, men	**1.78 [1.28;2.47] (<0.001)**	1.15 [0.83;1.58] (0.399)	**1.91 [1.49;2.46] (<0.0001)**	**1.91 [1.52;2.40] (<0.0001)**
Age, 10-y groups	**3.08 [2.65;3.61] (<0.0001)**	**1.67 [1.47;1.90] (<0.0001)**	**1.13 [1.04;1.23] (0.006)**	**1.81 [1.66;1.97] (<0.0001)**
Low education	1.17 [0.84;1.63] (0.351)	**1.62 [1.15;2.29] (0.006)**	**1.48 [1.12;1.95] (0.006)**	**1.67 [1.30;2.15] (<0.0001)**
High alcohol consumption	0.89 [0.62;1.27] (0.524)	1.37 [0.97;1.92] (0.076)	0.83 [0.62;1.10] (0.197)	0.89 [0.69;1.16] (0.402)
Current smoker	0.96 [0.63;1.47] (0.863)	0.82 [0.52;1.26] (0.380)	1.05 [0.8;1.43] (0.745)	0.93 [0.70;1.23] (0.608)

Table S4. Results of linear regression models to assess the relationship between the number of senses with reduced capacities and eating behavior. The explanatory variables are in rows and the response variables (food adventurousness and liking groups) in columns. Food adventurousness has been included in the model for liking groups. The values are betas, 95% confidence interval in square brackets, and p-value in round brackets. Significant results are indicated in bold.

	**Food Adventurousness**	**Alcoholic beverages**	**Cheeses**	**Fish**	**Fruit**	**Meat**	**Sweets**	**Vegetables**
Sex, male	-0.09 [-0.21;0.03] (0.135)	**1.44 [1.23;1.65] (<0.0001)**	-0.07 [-0.22;0.08] (0.359)	**0.45 [0.26;0.63] (<0.0001)**	**-0.22 [-0.35;-0-09] (<0.001)**	**0.51 [0.35;0.66] (<0.0001)**	**-0.38 [-0.55;-0.20] (<0.0001)**	**-0.43 [-0.57;-0.29] (<0.0001)**
Age, 10 years	**-0.05 [-0.09;-0.003] (0.035)**	**-010 [-0.18;-0.02] (0.0107)**	**0.07 [0.01;0.13] (0.0159)**	**0.17 [0.10;0.24] (<0.0001)**	0.03 [-0-02;0-08] (0.215)	**-0.08 [-0.14;-0-02] (0.006)**	-0.05 [-0.12;0.02] (0.166)	**0.22 [0.17;0.28] (<0.0001)**
Low education	**-0.34 [-0.48;-0.20] (<0.0001)**	**-0.33 [-0.57;-0.09] (0.008)**	-0.005 [-0.18;0.17] (0.96)	-0.18 [-0.39;0.03] (0.087)	-0.003 [-0.15;0.14] (0.971)	**0.29 [0.10;0.46] (0.0015)**	0.03 [-0.17;0.24] (0.736)	0.04 [-0.12; 0.20] (0.616)
Food adventurousness	-	**0.20 [0.10;0-30] (<0.0001)**	**0.24 [0.17;0.32] (<0.0001)**	**0.41 [0.32;0.50] (<0.0001)**	**0.17 [0.11;0.24] (<0.0001)**	0.07 [-0.001;0.14] (0.054)	**0.09 [0.008;0.18] (0.032)**	**0.23 [0.16;0.30] (<0.0001)**
Number of senses with reduced capacities	-0.08 [-0.15;0.0002] (0.0508)	**-016 [-0.29;-0.03] (0.0134)**	-0.06 [-0.15;0.04] (0.223)	**-0.12 [-0.23;-0.01] (0.0402)**	-0.06 [-0.14;0.02] (0.117)	0.04 [-0.06;0.13] (0.435)	-0.03 [-0.14;0.08] (0.552)	**-0.11 [-0.21;-0.03] (0.009)**
